# Decoding ecosystem heterogeneity and transcriptional regulation characteristics of multi-subtype renal cell carcinoma

**DOI:** 10.1016/j.heliyon.2024.e33196

**Published:** 2024-06-25

**Authors:** Kailong Xu, Jiang Li, LiWei Qin, Jie Liu, Heng Yang, Gang Dou, LiXin Ma, Yanming Dong, Yang Wang

**Affiliations:** a State Key Laboratory of Biocatalysis and Enzyme Engineering, School of Life Sciences, Hubei University, Wuhan, China; b College of Electrical Engineering and Automation, Shandong University of Science and Technology, Qingdao, China

**Keywords:** Renal cell carcinoma, Single-cell RNA sequencing, Macrophages, Cancer-associated fibroblasts, Cellular heterogeneity

## Abstract

**Background:**

Renal cell carcinoma (RCC) is characterized by its heterogeneity and the complexity of its tumor microenvironment. This study addresses the need to understand RCC at a cellular level, with a focus on its three main subtypes: clear cell (ccRCC), chromophobe (chRCC), and papillary renal cell carcinoma (pRCC).

**Objective:**

This study aims to comprehensively characterize the cellular diversity and intercellular communication networks of RCC subtypes using scRNA-seq technology. By focusing on macrophages and cancer-associated fibroblasts (CAFs), we seek to reveal their functional states, developmental trajectories, and signaling pathways.

**Methodology:**

We utilized single-cell RNA sequencing (scRNA-seq) data from various kidney cancer subtypes. Advanced analytical techniques, including Uniform Manifold Approximation and Projection (UMAP) and Reactome Gene Set Variation Analysis (ReactomeGSA), were employed to assess cellular heterogeneity and pathway activities. The developmental dynamics of macrophages were studied using CytoTRACE, and cell-to-cell communication was analyzed to identify subtype-specific interaction networks.

**Results:**

Our comprehensive analysis revealed significant cellular diversity within RCC. Distinct macrophage and CAF subpopulations were identified, each exhibiting unique gene expression profiles and pathway activities. Notably, ccRCC showed prominent bidirectional communication between macrophages and CAFs, while chRCC and pRCC displayed disrupted signaling pathways. Metabolic pathway analysis reflected the adaptability of macrophages and CAFs to the tumor microenvironment, and the MIF signaling pathway was identified as a key mediator of cellular interactions.

**Conclusion:**

The study highlights the cellular heterogeneity and the intricate communication networks within RCC subtypes, underscoring the complexity of the tumor microenvironment. Our findings suggest that targeting specific cellular interactions and pathways may offer new avenues for therapeutic intervention in RCC. The unique macrophage and CAF profiles across RCC subtypes provide valuable insights for the development of personalized and targeted treatment strategies.

## Graphical abstract

**Figure:**
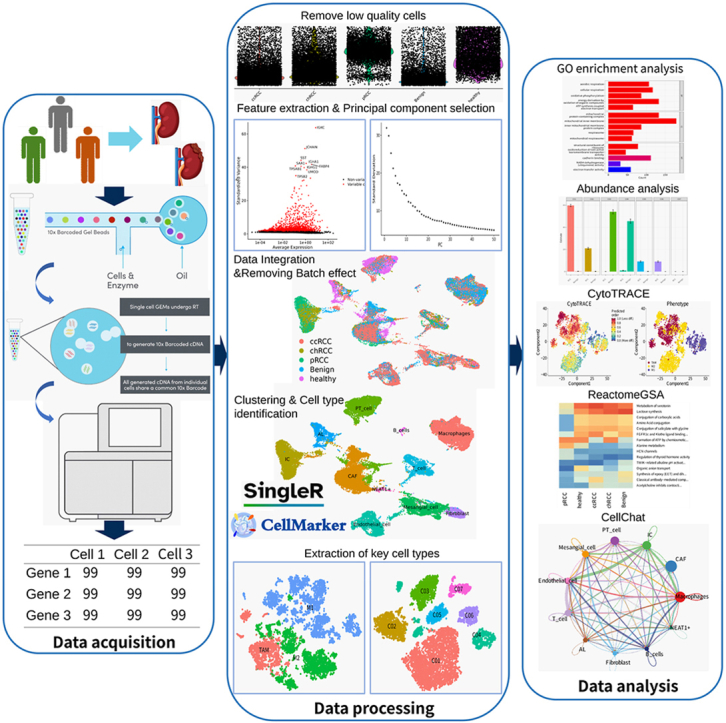

**Table:** 

Abrrevation	Full name
RCC	Renal cell carcinoma
UMAP	Uniform Manifold Approximation and Projection
T-SNE	t-Distributed Stochastic Neighbor Embedding
CAFs	cancer-associated fibroblasts
GO	gene ontology
BP; CC; MF	biological process; cell component; molecular function
TAMs	Tumor-associated macrophages
TME	Tumor micro-environment
NEAT1+ cell	Cells with high expression of gene NEAT1
ccRCC	clear cell Renal cell carcinoma
chRCC	chromophobe Renal cell carcinoma
pRCC	papillary Renal cell carcinoma
ReactomeGSA	Reactome Gene Set Variation Analysis
scRNA-seq	Single-cell RNA sequencing
ChIP-seq	Chromatin Immunoprecipitation sequencing
scTCR-Seq	single-cell T cell receptors sequencing
NCBI	National Center for Biotechnology Information
GEO	Gene Expression Omnibus data base
GEPIA	Gene Expression Profiling Interactive Analysis
TCGA	The Cancer Genome Atlas
IC	Intercalated cells
PT	Proximal Tubular cell
AL	Ascending Limb cells

**Simple Summary:** Renal cell carcinoma (RCC) is a highly heterogeneous kidney cancer, comprising distinct subtypes. Different subtypes displaying distinct pathological features and prognostic outcomes. In this research, our aim was to elucidate the cellular compositions, cell-type proportions, transcriptional regulation patterns, cell-cell communication, and differentiation trajectories associated with different RCC subtypes and their tissue origins, we performed single-cell transcriptome sequencing analyses of kidney tissue from healthy individuals and patients with RCC. We noticed healthy individuals exhibited a higher expression and transcriptional activity of metallothionein genes in kidney tissues compared to those with renal cell carcinoma. In addition, we observed similar “TAM polarization” processes in clear cell renal cell carcinoma (ccRCC) and chromophobe renal cell carcinoma (chRCC), setting them apart from papillary renal cell carcinoma (pRCC). By shedding light on the transcriptional regulation features and trajectory differentiations within different RCC subtypes or tissue states, our study contributes to a deeper understanding of the complex molecular landscape underlying RCC heterogeneity.

## 1 Introduction

Renal cell carcinoma (RCC) represents a group of heterogeneous malignancies, each characterized by distinct pathological features and clinical outcomes. The cellular complexity within the RCC tumor microenvironment plays a pivotal role in disease progression, response to therapy, and overall prognosis. Understanding this complexity is crucial for the development of more effective, personalized therapeutic strategies [1]. RCC is primarily classified into three major subtypes: clear cell renal cell carcinoma (ccRCC), the most common and aggressive form; chromophobe renal cell carcinoma (chRCC), known for its distinct histological features; and papillary renal cell carcinoma (pRCC), which presents in two different types. Each subtype harbors unique genetic alterations and microenvironmental interactions, contributing to their diverse clinical behaviors [2,3].

Recent advancements in single-cell RNA sequencing (scRNA-seq) technology have provided unprecedented insights into the cellular heterogeneity and intercellular communication within tumors [[4], [5], [6]]. This technology has emerged as a powerful tool for dissecting the complex cellular landscapes of cancers, including RCC. By enabling the analysis of individual cell types, scRNA-seq helps in delineating the roles of various cellular components, such as cancer cells, immune cells, and stromal cells, in tumor biology [7,8].

Among the key players in the RCC microenvironment are macrophages [9,10] and cancer-associated fibroblasts (CAFs) [11]. Macrophages, with their diverse phenotypes and functions, contribute significantly to tumor growth, metastasis, and immune evasion. CAFs, on the other hand, are instrumental in shaping the tumor stroma, facilitating angiogenesis, and modulating immune responses. The interactions between these cell types and cancer cells are complex and dynamic, often influencing treatment efficacy and patient outcomes.

This study aims to comprehensively characterize the cellular diversity and intercellular communication networks within RCC subtypes using scRNA-seq. By focusing on macrophages and CAFs, we seek to uncover their functional states, developmental trajectories, and signaling pathways. We hypothesize that these insights will not only enhance our understanding of RCC pathophysiology but also identify potential therapeutic targets within the tumor microenvironment. The differential cellular interactions and signaling pathways across RCC subtypes could offer novel avenues for targeted intervention, paving the way for improved patient-specific treatment strategies in renal cancer.

## 2 Materials and methods

Design of study:In this study, we delve into the complexity of renal cell carcinoma (RCC) by analyzing single-cell transcriptomic data from 11 RCC patients and 2 healthy individuals. We developed a comprehensive workflow from data quality control to integrative analysis to reveal the dynamic changes in RCC cellular heterogeneity and the tumor microenvironment. Through dimensionality reduction and clustering techniques, we successfully identified and annotated different cell types, providing a clear view of transcriptional regulation within the tumor microenvironment. Further downstream analyses, including the application of tools such as ReactomeGSA, CytoTRACE, and CellChat, enabled us to deeply understand the communication patterns between RCC cells, the trajectories of cellular development, and the activity levels of biological pathways.

### 2.1 Download and collation of data

The data for this topic are downloaded from the GEO database, data numbers GSE159115 [12], GSE152938 [13], and GSE171458 [14]. The data included 6 Benign adjacent samples (GSM4819727, GSM4819729, GSM4819730, GSM4819731, GSM4819734, GSM4819736), 7 ccRCC tumor parenchymal parts (GSM4819725, GSM4819726, GSM4819728, GSM4819733, GSM4819735, GSM4819737, GSM4630028), 2 chRCC tumor parenchymal tissues (GSM4819732, GSM4630030), 1 case of diseased pRCC tumor parenchymal parts (GSM4630027), and 2 healthy kidney tissues (GSM5225906, GSM5225907), a total of 18 samples.

### 2.2 Data analysis

#### 2.2.1 Data preprocessing and quality control

Seurat [15] R package was used for data processing and related analysis, and quality control parameters were set to filtrate high-quality cells for subsequent analysis, among which the threshold of gene number was set to 500–4000, mitochondrial gene <40 %, and ribosomal gene <30 %. The analysis software and program versions are shown in Supplementary Table 4.

#### 2.2.2 Dimensionality reduction clustering and cell type identification

The samples were integrate using anchors method in the R package “Seurat” and core cells were obtained by filtering scRNA-seq. Ineligible cells include genes that can only be detected in 3 or fewer cells and low-quality cells with less than 200 genes detected will be excluded from subsequent analysis. Gene expression of core cells was normalized using function SCTransform(.) [16], and then the top 2000 genes with highly variable characteristics were screened by ANOVA. Principal component analysis (PCA) was performed on single-cell samples, and the top 35 principal components (PC) were selected for subsequent analysis. The RunHarmony function [17] was used to eliminate the batch effect. The umap algorithm [18] was used to perform an overall dimensionality reduction analysis on the top 35 PC pairs of samples, the resolution was set to 0.3 during the clustering process. The FindClusters(.) function is used to cluster based on the SNN graph. The algorithm for modularity optimization we used is original Louvain [19] algorithm. The FindAllMarker function was used to identify the Marker gene of each cell subpopulation, using the R package “singleR [10]" package, HumanPrimaryCellAtlasData, and BlueprintEncodeData were used as reference data for auxiliary annotation, followed by the CellMarker database [20] and previous studies to find marker genes for manual annotation of different clusters.

#### 2.2.3 Functional enrichment analysis

In this project, two groups of objects were selected for differential gene identification and enrichment analysis: (1) for tumor sample Cancer-associated fibroblast clusters (CAF) in tissue and benign adjacent tissues; (2) Macrophage clusters targeting tumor parenchyma and benign neighboring tissues. Marker gene was taken between the two groups of data to obtain the differential gene set, and the significance threshold was set as p_val_adj<0.05, |avg_log2FC|>0.5. enrichment analysis of gene ontology (GO) [21] was performed on the differential genes identified in the 2 groups of data, including biological process (BP), cell component (CC) and molecular function (MF), and enrichment results were visualized using enrichment plot software package. With p < 0.05 as the critical value, it was considered statistically significant.

#### 2.2.4 Cell–cell communication analysis

The CellChat v1.6.1 software [22] was used to infer cell–cell communication based on ligand–receptor(L–R) interaction with default parameters. CellChat can quantitatively infer and analyze intercellular communication networks from scRNA-seq data and contains L–R interaction databases (http://www.cellchat.org/). For each L–R pair, only the secreted signaling interaction category was considered for downstream analysis. We filtered out the cell–cell communication if there are fewer than 10 cells in certain cell groups. The statistical sig-nificance of communication probability values was assessed by a permutation test. p < 0.05 was considered statistically significant. Function “computeCommunProbPathway” was employed to Infer the cell-cell communication at a signaling pathway level. Afterwards, we compare the signaling pathways that contribute most to the overall contribution of each L–R in cell populations from different samples and visualize all cells associated with this signaling pathway. This was done by setting the parameter signaling in the function “netVisual_chord_cell”.

#### 2.2.5 Subdivision of macrophages and CAF cell populations

In order to better analyze the interactions between different cell clusters, we extracted macrophages and CAF cell clusters respectively for dimensionality reduction clustering and grouping annotation. We selected n = 32 and n = 20 as the dimensionality reduction of macrophages and CAF, and set 0.3 and 0.7 as the clustering resolution, respectively, and used the T-SNE method to display the clustering results. For the parameter selection problem in CAFs and macrophage analysis, we applied multiple criteria to determine the best dimension reduction parameters such as n values and clustering resolution. These criteria include, but are not limited to, sample size and the expected diversity of cell types. We go through multiple validations and analyses to ensure that the selected parameters most accurately reflect the biology of the cell population. Using the freqCI function in R-package REdaS [23], we calculated confidence intervals for the relative frequencies of different T cell subtypes, macrophage subtypes, and CAF subtypes to assess their abundance changes.

#### 2.2.6 Single-cell trajectory analysis: macrophages in renal cell carcinoma

R package monocle2 [24] and CytoTRACE [25] were applied to conduct cellular trajectory analysis. The CytoTRACE method predicts the relative differentiation status of cells based on single-cell RNA sequencing data. Monocle 2 assumes that one-dimensional “time” can describe high-dimensional expression values, the so-called single-cell pseudo-time analysis. If the cell expression exceeds 1 %, the mean expression value is greater than 0.3, and the dispersion experience value is > 1, the genes used to sequence the cells are selected. Based on the “DDRTree” method, the data is reduced to two dimensions and the cells are then sequenced along the trajectory.

#### 2.2.7 Pathway enrichment analysis aimed at CAF and macrophages

To explore pathway differences between different cell clusters, we used the ReactomeGSA package [26] for pathway enrichment analysis based on the Reactome database [27]. ReactomeGSA can integrate and compare data from different species and different omics techniques, using the ssGSEA algorithm to calculate the activity score of each pathway. We analyzed the Seurat object using the analyse_sc_clusters function of the ReactomeGSA package, calculated the average gene expression for each cell cluster, and mapped it to the Reactome pathway. Paths with significant differences were screened out and visualized using the plot_gsva_heatmap function of the ReactomeGSA package.

## 3 Results

### 3.1 Characterization of renal cancer single-cell atlas and identification of gene expression features

**Fig. 1:**
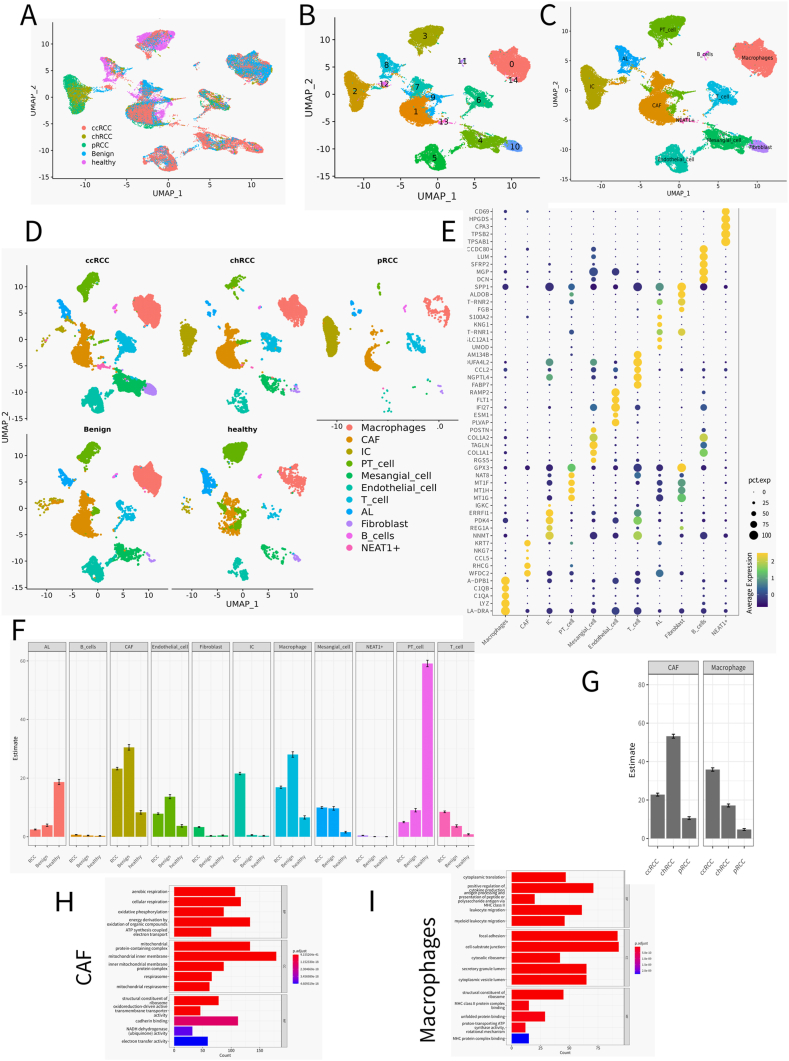
The immune landscape of patients with RCC at single-cell resolution. A)-D) UMAP embedding of transcriptional profiles from all samples. Each dot represents a single cell, and colors represent clusters denoted by inferred cell type or tissue type. E) The Dot plot shows the expression of Top5 Marker across each cell types F) Fractional changes for each cell type across the three Sample types. Error bars indicate the 95 % confidence interval for the calculated relative frequencies. ∗p < 0.01 using a chi-square test of independence. G) Fractional changes for Macrophages and CAF across the three Tumor subtypes. Error bars indicate the 95 % confidence interval for the calculated relative frequencies. ∗p < 0.01 using a chi-square test of independence. H)GO enrichment analysis of CAF differentially expressed genes in different tumors and Benign adjacent Sample. I)GO enrichment analysis of Macrophagesdifferentially expressed genes in different tumors and Benign adjacent Sample.

In the quest to decode the complexity of renal cancer at the cellular level, we embarked on an extensive analysis of single-cell RNA sequencing (scRNA-seq) data across three principal subtypes of kidney cancer: clear cell renal cell carcinoma (ccRCC), chromophobe renal cell carcinoma (chRCC), and papillary renal cell carcinoma (pRCC). The endeavor began with the integration of multi-source datasets, which necessitated meticulous batch correction to ensure the validity of cross-sample comparisons. Following rigorous quality control measures, a substantial cohort of 51,532 cells was curated for in-depth analysis. The refined dataset comprised a significant representation of tumor-derived cells, with 35,236 cells spanning across ccRCC, chRCC, and pRCC, alongside 8840 cells from adjacent benign tissues and 7456 from healthy kidney samples (S.Table1). Leveraging the Uniform Manifold Approximation and Projection (UMAP) technique, we achieved a sophisticated dimensionality reduction that facilitated unsupervised clustering of diverse cell types (Fig. 1A and B). This process was informed by a conjunction of subtype-specific gene expression data, existing biological databases, and the current scientific literature, allowing for the precise annotation and characterization of the cellular constituents within the renal cancer milieu (Fig. 1C–E). We have distinguished NEAT1+ cells separately, because they represent a novel and interesting cell population that has not been reported before in RCC. NEAT1 is a long non-coding RNA that is involved in the formation and maintenance of nuclear paraspeckles, which are implicated in various biological processes, such as gene expression regulation, stress response, and tumorigenesisNEAT1 has been shown to have oncogenic properties in several solid tumors, such as prostate cancer [28], gastric cancer [29], and gliomaHowever [30].

Quantitative assessment of the cellular landscape revealed a pronounced abundance of CAFs and macrophages in ccRCC, contrasting with the chRCC and pRCC samples, where endothelial and immune cells were more prevalent (Fig. 1 F and G). This variance underscores the intrinsic heterogeneity of the microenvironment across kidney cancer subtypes and highlights the distinct cellular dynamics that potentially influence tumor behavior and immune interactions. Delving into the gene expression profiles of these cellular clusters, we identified unique expression patterns that demarcate the cell types. CAFs, for instance, were distinguished by their overexpression of genes linked to extracellular matrix remodeling—a key process in tumor progression. Macrophages, on the other hand, displayed expression signatures indicative of their role in immune regulation, which may have critical implications for the tumor's immune microenvironment (Fig. 1H and I).

The gene set enrichment analysis further accentuated this distinction, revealing an enrichment of pathways in CAFs that are integral to tumor proliferation, migration, and invasion, including the TGF-β signaling and collagen-related pathways. Contrastingly, macrophages showed an enrichment of pathways associated with inflammatory responses and cytokine activity, reflecting the nuanced immune modulation mechanisms at play within the tumor microenvironment. Together, these findings shed light on the intricate cellular tapestry of kidney cancer, illuminating the specialized roles that various cell types, particularly CAFs and macrophages, may play in shaping the tumor landscape and modulating the immune response. This comprehensive single-cell atlas not only serves as a valuable resource for understanding the cellular heterogeneity of kidney cancer but also provides a foundation for potential therapeutic strategies aimed at manipulating the tumor microenvironment for clinical benefit.

### 3.2 Dissecting the macrophage landscape in renal carcinomas

**Fig. 2:**
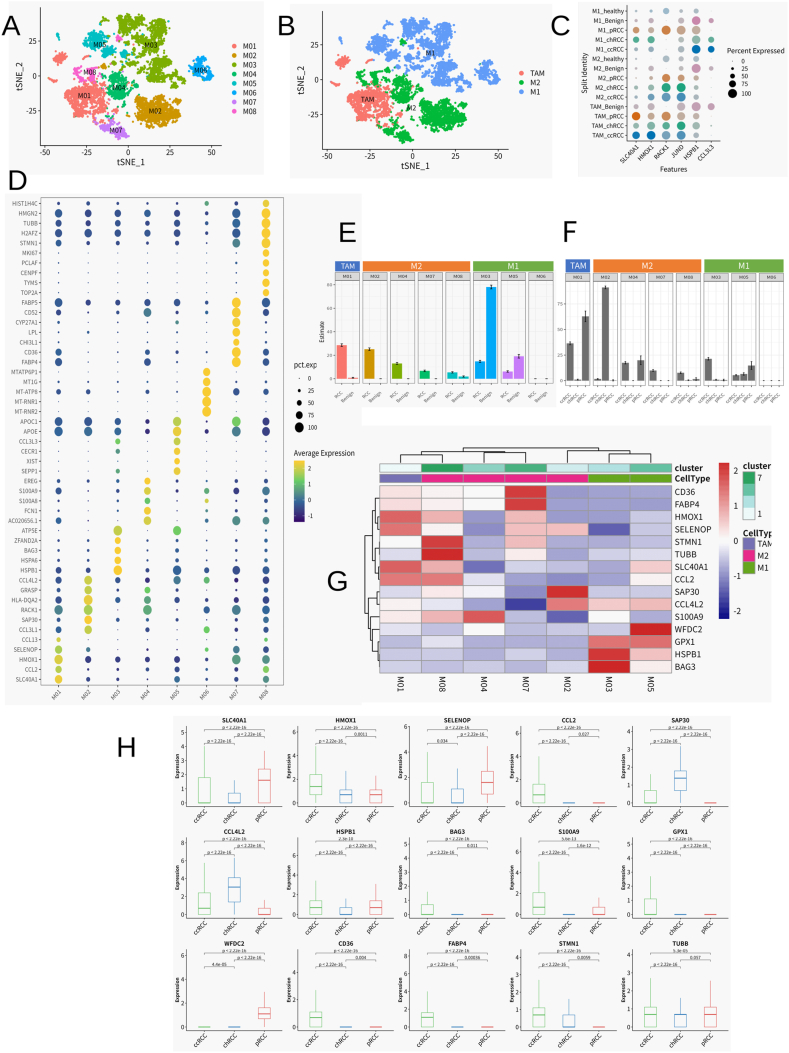
The Macrophage landscape of renal cell carcinoma A) Dimensional reducing and clustering on the data for Macrophages B)Add annotation for Macrophage clustering results. TAM:tumor-associated macrophages; M1; M2 C)Dotplot of Top2 Marker in 3 Macrophage subtypes (by origion) D) The Dot plot shows the expression of Top5 Marker across 8 Macrophage clusters E) Fractional changes for each Macrophage subtype across the Tumor and Benign adjacent Sample. Error bars indicate the 95 % confidence interval for the calculated relative frequen-cies. ∗p < 0.01 using a chi-square test of independence. F) Fractional changes for each Macrophage subtype across the three Tumor subtypes. Er-ror bars indicate the 95 % confidence interval for the calculated relative frequencies. ∗p < 0.01 using a chi-square test of independence. G) Heatmap of normalized Macrophage marker expression for 7 Macrophage clusters from tumor sample. I)Box plots of the expression levels of the genes in Figure G in three renal cancer types, with each box plot representing the expression level of a gene across different renal cancer type. Kidney cancer types were marked on the horizontal axis, and gene expression levels were marked on the vertical axis. The *P*-value is calculated using T. TEST to check whether there are significant differences in the expression levels of the same gene in different cell types.

Our exploration into renal carcinoma's cellular architecture through single-cell transcriptomics has unveiled distinctive macrophage phenotypes dispersed across normal, benign adjacent, and various malignant kidney tissues, including those from ccRCC, chRCC, and pRCC (Fig. 2A and D). This analysis afforded us a panoramic view of macrophage localization and density within the renal ecosystem. Subsequent annotation of macrophage subsets in Fig. 2B has refined our grasp of their transcriptional profiles, delineating their specialized functions in the context of renal cancer. We categorized the macrophages into three primary classes: (1) the classically activated M1 subtype, hallmarked by elevated CD86 and IL1B expression; (2) the alternatively activated M2 group, distinguished by MRC1 and CD163 expression; and (3) the tumor-associated macrophages (TAMs), identified by CCL2 and PDGFB expression [9]. Notably, genes such as SLC40A1 and HMOX1 were significantly upregulated in TAMs, whereas RACK1 and JUND were predominantly expressed in M2 macrophages, with HSPB1 being a marker of the M1 phenotype (Fig. 2C).

The varied expression ratios of TAMs, M1, and M2 macrophages across renal tissue types (Fig. 2E), their distribution across RCC subtypes (Fig. 2F–S.Table2), and the heatmap illustrating subset-specific gene overexpression (Fig. 2G) together unravel the complex fabric of the tumor environment. These macrophage-specific gene expression profiles underscore their divergent contributions to renal cancer's pathology and pave the way for novel therapeutic targets. Boxplot analyses underscored these transcriptional variations, exposing significant disparities in the expression levels of stress response genes such as SELONP and STUB1 across different cancer subtypes (Fig. 2H), highlighting the influence of macrophage-driven mechanisms on tumor dynamics and clinical prognosis.

While the sheer quantity of macrophages in RCC offers limited prognostic utility, the predominance of the M2 phenotype, or a higher M2/M1 ratio, has been consistently linked with adverse outcomes [31]. The immunosuppressive functions of M2 macrophages within RCC, characterized by their expression of PD1 ligands and secretion of IL-10, play a pivotal role in modulating T cell responses. The production of IL-23 by M2 macrophages, promoting the expansion of regulatory T cells, further compounds the immune suppression within the tumor milieu [32]. A deeper understanding of the enrichment of M2 macrophages and the dynamics of functional T cells during tumor progression is essential for dissecting RCC's immunosuppressive environment. In sum, our in-depth analysis has illuminated the distinct macrophage subpopulations within RCC and their unique distributions, enhancing our comprehension of the intricate interplay between immune cells and the tumor microenvironment. These insights are instrumental in guiding the development of targeted immunotherapy approaches for RCC.

### 3.3 Tracing macrophage developmental dynamics in RCC

**Fig. 3:**
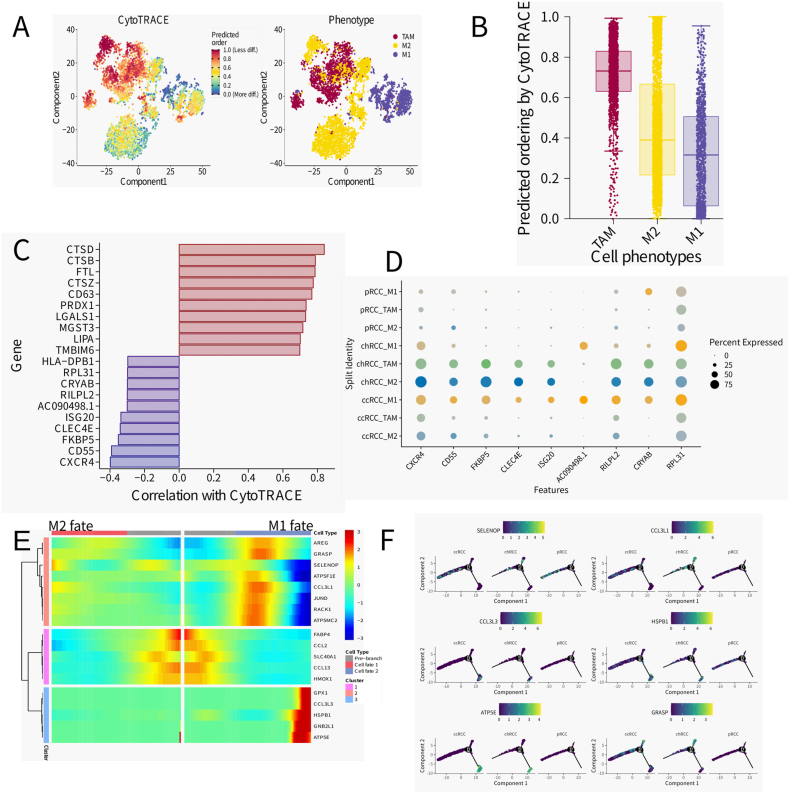
Subtypes and development trajectory of Macrophage A) Application CytoTRACE to dissect Macrophage differentiation B) Boxplots showing CytoTRACE values for 3 Macrophage subtype C)The 10 genes with the highest positive and negative correlation with CAF development were calculated by Cytotrace, The horizontal axis shows the correlation, the vertical axis shows the names of the genes, and the colors of the bar chart are red and purple, respectively, indicating positive and negative correlations. D)The 10 genes negatively correlated with cell development in C) were displayed in dotplot to check their expression in specific cell clusters across 3 different tumor samples G) BEAM analysis in Monocle2 indicates different expression patterns along the development of Macrophage to different Macrophage subtype fate. H) Relative expression of GNLY, KLRD1,COL1A2,HLA-DQA2, CMC1 and GZMK in Macrophage trajectory branches.

In the furtherance of our inquiry into the macrophage milieu of renal cancer, we embarked on an investigation into the evolutionary pathways and functional profiles of various macrophage subpopulations. Employing CytoTRACE [25], a tool that prognosticates cellular developmental statuses through the lens of gene expression complexity, we delineated a developmental continuum, as captured in Fig. 3A. This continuum, represented in a bidimensional scatterplot, color-codes cells according to their developmental score, highlighting a progression from nascent to fully differentiated states. Moreover, the analysis juxtaposed these developmental scores against macrophage phenotypes—TAM, M2, and M1—melding the notion of cellular evolution with functional differentiation. In a quantitative display, Fig. 3B unpacks this predicted ordering by CytoTRACE, showcasing a diverse array of developmental scores across these phenotypes. Such diversity underscores the transcriptional richness within the macrophage contingents, alluding to a spectrum of cellular maturity and task-specific specialization.

Fig. 3C takes this analysis a step further by correlating distinct genes with their respective CytoTRACE scores to unearth potential drivers behind the observed differentiation of macrophages. Genes such as CTSD and CTSC, exhibiting a strong positive correlation, are implicated in macrophage maturation. Conversely, genes like CXCR4, inversely correlated, hint at a role in sustaining an undifferentiated or progenitor state. Dot plot analysis in Fig. 3D corroborates these gene expressions across macrophage subsets within distinct renal cancer subtypes, affording us a granular view of the gene expression signatures that hallmark TAM, M2, and M1 cells in ccRCC, chRCC, and pRCC. Such insights shed light on the potential influence of RCC subtypes on macrophage functionality and identity. Advancing to Fig. 3E–a heatmap serves to illustrate the correlation between gene expression and macrophage destinies, M1 and M2. The heatmap's color intensity signals the strength of these correlations, providing an at-a-glance synopsis of genes that track alongside macrophage polarization. Complementarily, Fig. 3F presents a suite of line plots that trace the gene expression trajectories across dimensionality-reduced components, underscoring the link between gene expression trends and macrophage differentiation trajectories within the renal cancer framework.

Collectively, these multifaceted analyses have unraveled the transcriptional intricacies that orchestrate macrophage differentiation and functionality in renal cancer. The genes we've identified, along with their correlations to macrophage states, significantly bolster our comprehension of the tumor microenvironment's complexities and proffer promising targets for therapeutic strategies that aim to recalibrate macrophage activity within the cancerous landscape.

### 3.4 Dissecting the signaling landscape of macrophage subsets in renal tissues

**Fig. 4:**
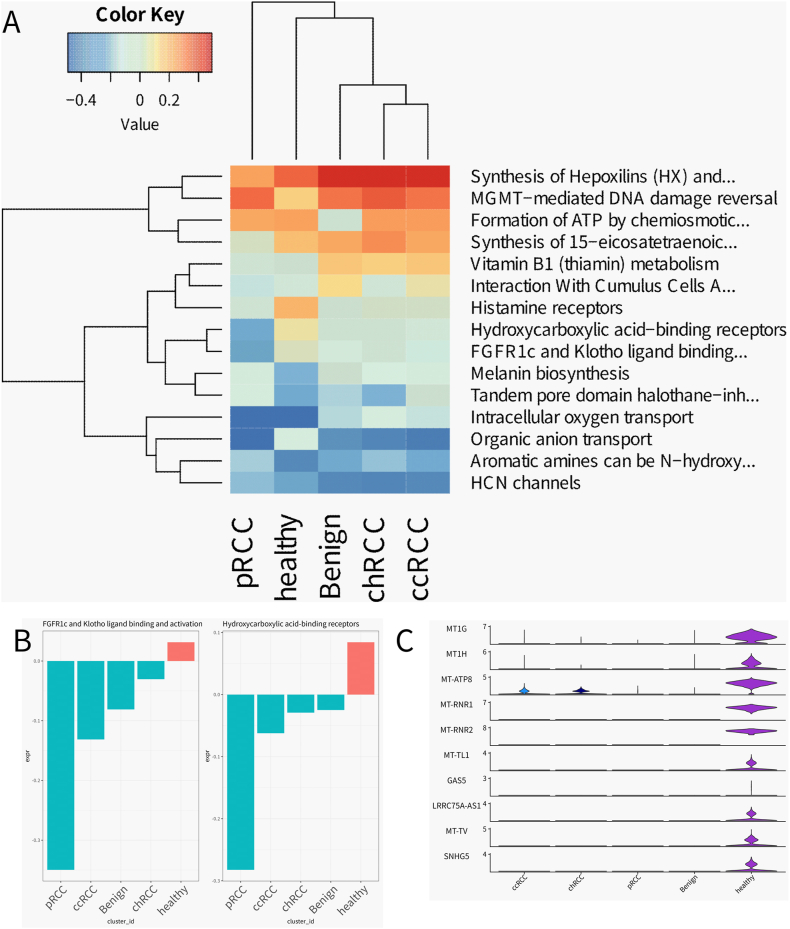
Pathway Analysis of Macrophage from all kidney sample. A) ReactomeGSA gene set variation based pathway-level expression in the identified Macrophage. Expression values were z-score normalized by pathway. B) Barplot generated by ReactomeGSA showing the activity of the “FGFR1c and Klotho ligand binding and activation” and “Hydroxycarboxylic acid-binding receptors” pathway in different samples. The horizontal axis shows the sample grouping, the vertical axis represents the expression value of the path, and the color represents the positive or negative expression value. C) To compare the expression of differentially expressed genes in macrophages of healthy samples in all cell clusters.

In an endeavor to decode the functional landscapes of macrophage subsets within renal tissue spectrums, our study engaged in a comprehensive pathway analysis. This analysis spanned healthy, benign, and neoplastic renal samples, employing Reactome Gene Set Variation Analysis (ReactomeGSA) to normalize gene expression across the macrophage populations by pathways, applying z-score transformations for cross-sample comparability (Fig. 4A). The resulting heatmap vividly details the relative pathway activities, with clusters revealing the extent of activation or suppression of pivotal cellular functions, thereby delineating the distinct physiological roles shaped by the microenvironment. The precision of ReactomeGSA facilitated the generation of bar plots (Fig. 4B and C), which brought into focus the activities of specific pathways, notably “FGFR1c and Klotho ligand binding and activation” and “Hydroxycarboxylic acid-binding receptors.” These visualizations laid bare the disparate engagement of these pathways across macrophage subsets, offering a window into their potential functional differentiation within the context of renal disease. Our findings illuminated the intricate network of signaling pathways that operate within macrophages, manifesting a spectrum of activity that is not merely reflective of health or disease states but is also finely modulated across the varied subtypes of kidney cancer. This heterogeneity in pathway utilization highlights the macrophages' capacity to adapt and respond to the nuances of the tumor microenvironment.

The comprehensive nature of this analysis provides an overarching view of the operational states of macrophages in renal contexts. The distinct signatures of pathway activity we uncovered are emblematic of the diverse roles macrophages undertake, ranging from maintaining renal homeostasis to engaging in the oncogenic process. The elucidation of these pathways deepens our grasp of the multifunctional roles of macrophages within renal health and pathology and may inform the development of targeted therapeutic interventions aiming to recalibrate the immune milieu in the face of renal cancer.

**Fig. 5:**
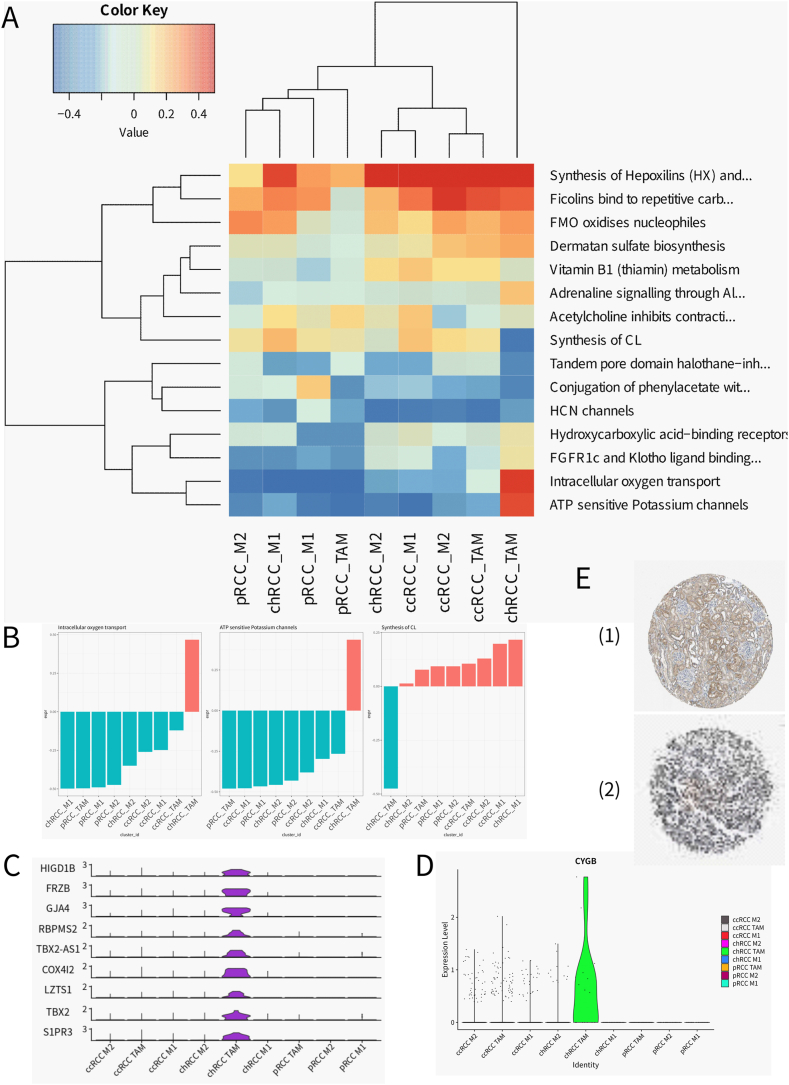
Analysis of macrophage subtype pathways in different tumor samples. A) ReactomeGSA gene set variation is expressed at pathway level in macrophage subtypes across different tumor samples. The expression values were normalized by z-score by path. B) Bar graphs generated by ReactomeGSA show the activity of the “Intracellular oxygen transport” “ATP sensitive Potassium channels” and “Synthesis of CL” pathway in macrophage subtype across different tumor samples. The horizontal axis represents the sample grouping, the vertical axis represents the expression value of the path, and the color represents the expression value of the positive or negative. C) To compare the expression of differentially expressed genes in TAM cells of chRCC samples in all cell clusters D) Expression of CYGB in all cell clusters E)Expression of CYGB in (1)normal tissue sections and (2)tumor pathological tissue sections.

To chart the intricate functional terrain of macrophage subsets within renal cell carcinoma (RCC), we undertook a meticulous pathway analysis. This endeavor shed light on the diverse signaling pathways operative within distinct macrophage identities under varying conditions, specifically within clear cell (ccRCC), chromophobe (chRCC), and papillary (pRCC) subtypes. The resulting heatmap (Fig. 5A) revealed the z-score normalized pathway activities, casting a spotlight on the functional state variances among macrophage subsets across different renal tumors. This nuanced mapping provided a holistic view of pathway engagement, underscoring the molecular agility of macrophages in response to the distinctive demands of the tumor microenvironment.

The subsequent bar graphs (Fig. 5B) brought into sharp relief the variable activities of pivotal metabolic pathways, including “Intracellular oxygen transport,” “ATP-sensitive Potassium channels,” and “Synthesis of CL,” across the macrophage subtypes. These insights into the metabolic landscape of macrophages suggest a marked functional diversity that mirrors their roles within tumor biology and the shaping influence of the surrounding microenvironment. Our focused examination (Fig. 5C) delved into the gene expression profiles of TAM cells in chRCC samples, unearthing transcriptional signatures that may delineate the operational nuances of this particular macrophage subset in the chRCC setting. Furthermore, the expression trajectory of the Intracellular oxygen transport pathway associated gene CYGB across all cell clusters (Fig. 5D) and its detection results in pathological tissue slices of different samples (Fig. 5E) offered a glimpse into the gene's overarching involvement in renal cancer, with its distribution among cell types providing a window into the hypoxia-induced response and metabolic recalibrations of macrophages within the renal tumors.

This stratified analysis not only deepened our comprehension of the multifaceted roles played by macrophage subsets in renal cancer but also accentuated the potential utility of these cells as both biomarkers and therapeutic targets. The delineated pathway activities and gene expression patterns underscore the functional heterogeneity of macrophages, paving the way for more personalized and finely tuned therapeutic strategies in the management of RCC.

### 3.5 CAF subtype profiles and their implications in RCC

**Fig. 6:**
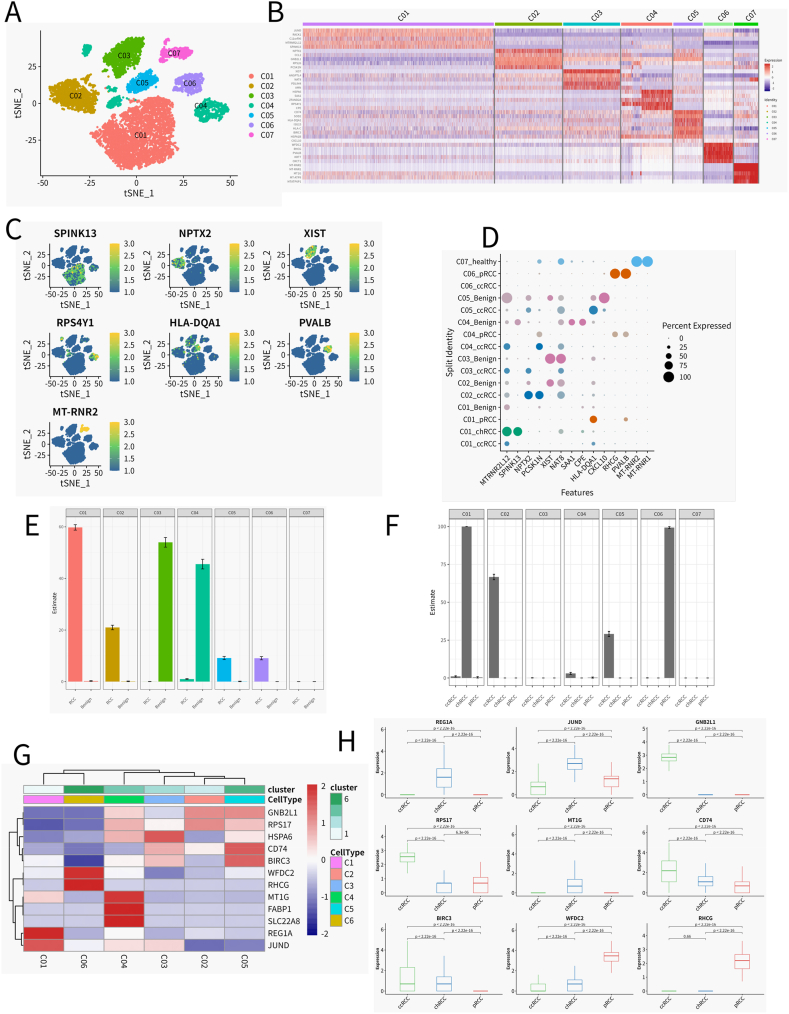
The CAF landscape of renal cell carcinoma A) Dimensional reducing and clustering on the data for CAF B) The Heatmap shows the expression of Top5 Marker across 7 CAF clusters C) t-SNE plots of Top1 Marker in 7 CAF clusters D)Dotplot of Top2 Marker in 7 CAF clusters (by origion) E) Fractional changes for each CAF subtype across the Tumor and Benign adjacent Sample. Error bars indicate the 95 % confidence interval for the calculated relative frequen-cies. ∗p < 0.01 using a chi-square test of independence. F) Fractional changes for each Macrophage subtype across the three Tumor subtypes. Error bars indicate the 95 % confidence interval for the calculated relative frequencies. ∗p < 0.01 using a chi-square test of independence. G) Heatmap of normalized CAF marker expression for 6 CAF clusters in tumor sample H)Box plots of the expression levels of the genes in Figure G in three renal cancer types, with each box plot representing the expression level of a different gene across different renal cancer type. Kidney cancer types were marked on the horizontal axis, and gene expression levels were marked on the vertical axis. The *P*-value is calculated using T. TEST to check whether there are significant differences in the expression levels of the same gene in different cell types.

Our inquiry into RCC encompassed an in-depth analysis of the cancer-associated fibroblast (CAF) spectrum within the tumor microenvironment. Dimensionality reduction techniques facilitated the identification of seven discrete CAF clusters, each bearing distinct transcriptional fingerprints (Fig. 6A). Such delineation of CAF subtypes highlights the intricate cellular composition within the RCC stroma. A heatmap (Fig. 6B) offered a visualization of the varied expression of the top five markers, accentuating the phenotypic heterogeneity intrinsic to CAFs. Further elucidated through t-SNE plots (Fig. 6C) and dot plots (Fig. 6D), we observed the defining expression patterns that demarcate these clusters, spotlighting the nuanced molecular signatures that typify each subgroup. Our investigation into the distribution of CAF subtypes within tumor and adjacent benign renal tissues underscored notable fractional disparities. The statistical analysis showcased in Fig. 6E validated these disparities as significant, hinting at the propensity of certain CAF subtypes to associate preferentially with either tumorigenic or benign regions, a factor that could profoundly influence tumor pathophysiology and stromal interactions.

Expanding this comparative lens to macrophage subsets across three RCC subtypes (Fig. 6F–S.Table3) unveiled distinct distribution patterns, indicative of the varied immune landscapes characteristic of each RCC variant. This heterogeneity in immune composition could be pivotal in modulating tumor dynamics and shaping therapeutic outcomes. The consolidated expression heatmap (Fig. 6G) mapped the transcriptional terrain of the CAF clusters, while subsequent evaluations of gene expression across diverse RCC types (Fig. 6H) unearthed significant inter-type variations, as corroborated by T. TEST. These variations signal distinct transcriptional landscapes prevalent in the renal cancer milieu.

In summation, our comprehensive portrayal of the CAF landscape within RCC intimates a significant role for fibroblast heterogeneity and its interplay with neoplastic cells in influencing disease evolution. The detailed profiling of CAF subpopulations presents a promising avenue for therapeutic intervention and prognostication, underscoring the potential of fibroblast diversity as a beacon for tailored treatment strategies and clinical outcome prediction in RCC.

### 3.6 CAF pathway profiling reveals metabolic and regulatory diversity in renal cancer

**Fig. 7:**
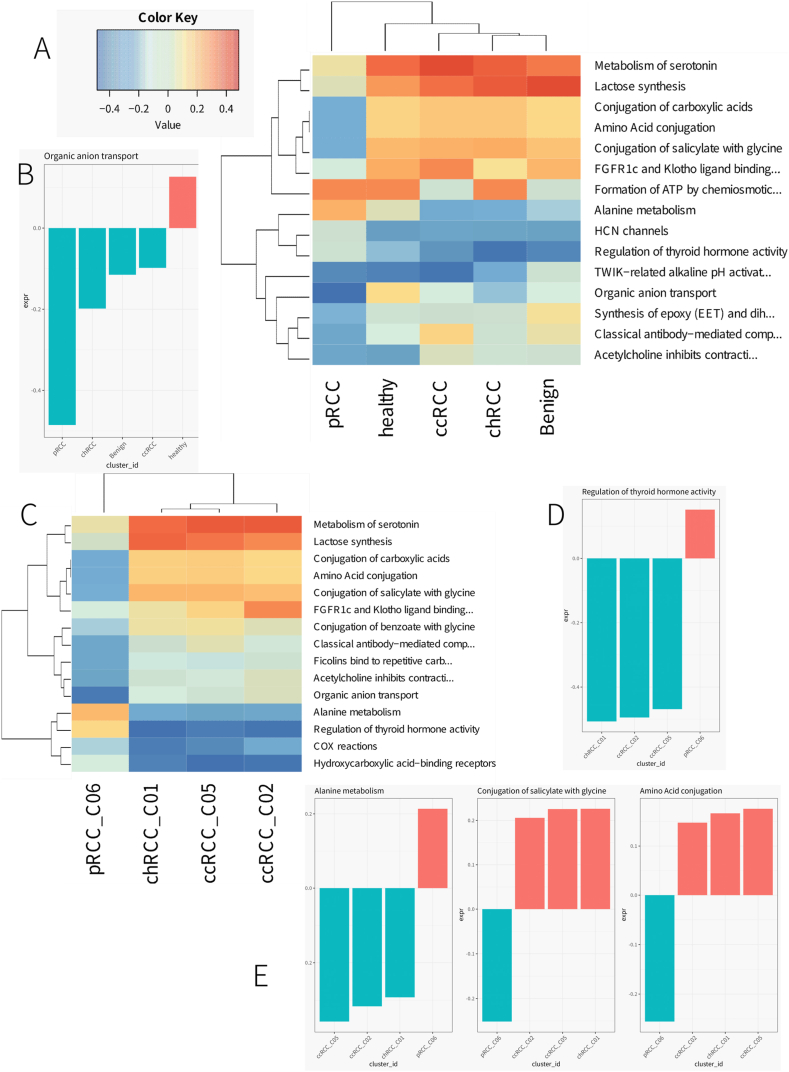
Pathway Analysis of CAF from all kidney sample. A) ReactomeGSA gene set variation based pathway-level expression in the identified CAF. Expression values were z-score normalized by pathway. B) Barplot generated by ReactomeGSA showing the activity of the “Organic anion transport” pathway in CAF across different samples. The horizontal axis shows the sample grouping, the vertical axis represents the expression value of the path, and the color represents the positive or negative expression value. C) ReactomeGSA gene set variation based pathway-level expression in the CAF cluster across different tumor samples. Expression values were z-score normalized by pathway. D) -E) Barplot generated by ReactomeGSA showing the activity of the “Regulation of thyroid hormone activity","Alanine metabolism","Conjugation of salicylate with glycine” and “Amino Acid conjugation” pathway in CAF cluster across different tumor samples. The horizontal axis shows the sample grouping, the vertical axis represents the expression value of the path, and the color represents the positive or negative expression value.

Through the deployment of Reactome Gene Set Variation Analysis (ReactomeGSA), we have meticulously profiled the pathway-level expressions of cancer-associated fibroblasts (CAFs) harvested from a diverse collection of kidney samples, as delineated in Fig. 7A. The z-score normalization of expression values has illuminated distinct pathway activities, mapping out a robust functional framework that characterizes CAFs within varied renal contexts. Our analytical foray into the CAF landscape revealed a pronounced variability in the “Organic anion transport” pathway among the samples, as depicted in Fig. 7B. The engagement level of this pathway indicates a metabolically diverse state of CAFs, which may be intricately linked to their roles in tumor biology and cellular crosstalk within the renal cancer microenvironment.

Probing deeper into the array of CAF clusters across different tumor samples, our study pinpointed key metabolic and regulatory pathways that exhibit differential activation, suggesting a bespoke functional imprint of CAFs contingent on the tumor environment (Fig. 7C). The subsequent bar plots (Fig. 7D and E) cast a spotlight on the activities of “Regulation of thyroid hormone activity,” “Alanine metabolism,” “Conjugation of salicylate with glycine,” and “Amino Acid conjugation” pathways. These pathways further unravel the metabolic versatility and potential regulatory influence exerted by CAFs across distinct tumor backdrops. The comparative expression levels across these pathways mirror the dynamic interactions between CAFs and the tumor microenvironment, with possible implications for oncogenesis and therapeutic responsiveness.

This granular pathway analysis of CAFs underscores the functional multiplicity of these fibroblasts within the renal carcinoma setting. The pathways brought to the fore in this study not only shed light on the intricate roles of CAFs, spanning from metabolic processes to hormonal regulation but also highlight their potential to significantly sculpt the tumor architecture. Such insights may pave the way for innovative therapeutic avenues aimed at modulating the fibroblast-driven facets of renal cancer pathology.

### 3.7 Unraveling cell communication dynamics in RCC subtypes

**Fig. 8:**
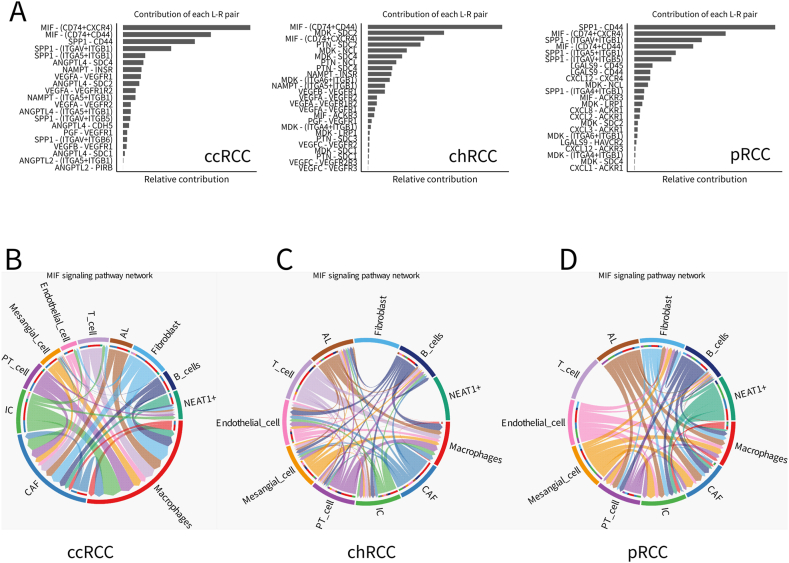
Global analysis cell-cell interaction features in different renal cell carcinoma subtypes. A)The contribution of each ligand-receptor to the overall signaling pathway from the different renal cancer samples was calculated and ranked separately. B) visualizing the cell-cell communication mediated by MIF (where each sector in the chord diagram is a ligand, receptor or signaling pathway).

Our study traverses the complex terrain of renal cell carcinoma (RCC) subtypes, revealing a comprehensive map of cell-to-cell interactions that define the unique microenvironments of clear cell (ccRCC), chromophobe (chRCC), and papillary renal cell carcinoma (pRCC). Fig. 8A unveils the intricate web of ligand-receptor (L-R) pair interactions within these subtypes, showcasing distinct communication patterns that underscore the diverse signaling landscapes of each subtype. In ccRCC, for instance, dominant interactions such as MIF - CD74 + CD44 and SPP1 - CD44 emerge, while chRCC and pRCC display varied pairings like CXCL9 - CXCR3, suggesting subtype-specific communication networks active within these tumors. Fig. 8 B-D provide a deeper dive into the MIF signaling pathway network across RCC subtypes, employing chord diagrams to visualize the extensive network of interactions among various cell types, including endothelial cells, fibroblasts, T cells, B cells, NEAT1+ cells, and macrophages. Notably, the ccRCC network reveals a robust interaction spectrum, particularly involving fibroblasts and macrophages, pointing to their potential roles in influencing tumor growth and immune responses. Contrastingly, chRCC and pRCC networks exhibit distinct interaction patterns, mirroring the unique tumor biology and microenvironment inherent to these subtypes.

In the realm of ccRCC, our analysis spotlights the pivotal communication between cancer-associated fibroblasts (CAFs) and macrophages as a key driver within the tumor microenvironment. This bidirectional signaling hints at their critical roles in shaping tumor dynamics and immune evasion mechanisms. In contrast, chRCC and pRCC subtypes show a notable absence of CAF-to-macrophage communication, indicating a disruption or reconfiguration of signaling pathways typically observed in ccRCC, suggesting a fundamental shift in the tumor microenvironment's architecture with potential impacts on disease progression and therapeutic outcomes. Fibroblast cells in chRCC are particularly noteworthy for their lack of interaction with other cell types, potentially signaling a unique aspect of chRCC tumor biology. This isolation could imply either a divergence in signaling activities or undetected communication patterns. Similarly, pRCC is characterized by a distinct lack of T cell interactions with other cells, possibly indicating an immune-suppressed or excluded phenotype within this subtype, which could be a contributing factor to its distinct clinical behavior compared to other RCC subtypes.

These findings paint a detailed picture of a complex, subtype-specific network of cellular interactions within RCC, shedding light on the nuances of tumor biology and paving the way for the development of targeted therapeutic strategies. The observed variations in cell-to-cell communication, particularly involving CAFs, macrophages, fibroblasts, and T cells, underscore the potential for targeting these interactions to disrupt the signaling networks driving tumor progression and immune modulation in RCC.

## 4 Discussion

Our comprehensive investigation into renal cell carcinoma (RCC) subtypes has unveiled a rich tapestry of cellular interactions, marked by distinct communication networks within the tumor microenvironment. This study, delving into the cell-to-cell dynamics of clear cell (ccRCC), chromophobe (chRCC), and papillary renal cell carcinoma (pRCC), highlights the diverse signaling landscapes that characterize each subtype, offering valuable insights into their unique pathological profiles. The differential ligand-receptor (L-R) pair interactions, as visualized in our analysis (Fig. 8A), underscore the heterogeneity of communication patterns across RCC subtypes. Notably, the dominant interactions observed in ccRCC, such as MIF - CD74 + CD44 and SPP1 - CD44, contrast sharply with the varied pairings in chRCC and pRCC. This observation suggests that each RCC subtype harbors a unique set of cellular dialogues, potentially driving the differences in tumor behavior and patient responses to treatment.

Our study's chord diagrams (Fig. 8B, C, and 8D) reveal the intricate network of interactions within the MIF signaling pathway, emphasizing the crucial roles of fibroblasts and macrophages in ccRCC. An important cellular component of the tumor microenvironment is cancer-associated fibroblasts (CAFs), which are a class of activated cells Interstitial cells are highly heterogeneous and plastic [11,33]. CAFs can secrete a variety of cytokines and chemotaxis Factors, growth factors, matrix metalloproteinases, etc. regulate tumor angiogenesis, tumor cell proliferation, invasion, migration Migration and stem cell properties inhibit the function of immune cells and promote tumor development and drug resistance [34,35]. The robust set of interactions involving these cell types indicates their potential influence on tumor growth and modulation of immune responses. Conversely, the unique interaction patterns in chRCC and pRCC reflect the distinct tumor biology and microenvironment of these RCC subtypes.

Particularly striking is the absence of communication from CAFs to macrophages in chRCC and pRCC, contrasting with the bidirectional signaling between these cell types in ccRCC(S Fig. 2). This finding suggests a potential disruption or reorganization of the signaling pathways typically active in ccRCC, hinting at a fundamental shift in the tumor microenvironment's architecture. Such a shift could significantly impact the progression and treatment response of chRCC and pRCC subtypes. The isolation of fibroblast cells in chRCC and the lack of T cell interactions in pRCC further accentuate the subtype-specific nature of RCC. The absence of T cell communication in pRCC, in particular, might indicate a suppressed immune response or an immune-excluded phenotype, contributing to the distinct clinical behavior of this subtype.

Collectively, our findings highlight the complexity of the RCC tumor microenvironment, with significant implications for the development of targeted therapies. The observed differences in cell-to-cell communication, especially the roles of CAFs, macrophages, fibroblasts, and T cells, offer potential targets for disrupting the signaling networks underpinning tumor progression and immune modulation. These insights underscore the need for personalized treatment approaches, taking into account the unique cellular interactions and signaling pathways of each RCC subtype. Future research should focus on exploring these targets further, paving the way for novel therapeutic strategies that can more effectively address the diverse challenges presented by RCC.

## Funding

This work was supported by the 10.13039/501100017589Hubei University Fund (202110703000002), Open Funding Project of the 10.13039/501100019937State Key Laboratory of Biocatalysis and Enzyme Engineering (SKLBEE20220021, SKLBEE20230024) and Knowledge Innovation Program of Wuhan-Shuguang Project (2022020801020325).

## Data availability statement

All the data used in this study were sourced from publicly available databases and published academic research, and are fully open for sharing. The datasets analyzed during the current research are available in the NCBI GEO repository under the following accession numbers: GSE159115, GSE152938, and GSE171458. The specific dataset files within these series include GSM4819727, GSM4819729, GSM4819730, GSM4819731, GSM4819734, GSM4819736, GSM4819725, GSM4819726, GSM4819728, GSM4819733, GSM4819735, GSM4819737, GSM4630028, GSM4819732, GSM4630030, GSM4630027, GSM5225906, and GSM5225907.

## CRediT authorship contribution statement

**Kailong Xu:** Formal analysis, Writing - original draft, Writing – review & editing. **Jiang Li:** Formal analysis, Writing - original draft, Writing – review & editing. **LiWei Qin:** Formal analysis, Writing - original draft, Writing – review & editing. **Jie Liu:**Software, Writing - original draft, Writing – review & editing. **Heng Yang:** Software, Writing - original draft, Writing – review & editing. **Gang Dou:** Validation. **LiXin Ma:** Conceptualization, Writing - original draft, Writing – review & editing. **Yanming Dong:** Conceptualization, Supervision, Writing – review & editing. **Yang Wang:** Conceptualization, Supervision, Writing – review & editing.

## Declaration of competing interest

The authors declare that they have no known competing financial interests or personal relationships that could have appeared to influence the work reported in this paper.
