# Supplementary material for: Decoding ecosystem heterogeneity and transcriptional regulation characteristics of multi-subtype renal cell carcinoma
Source: Heliyon. 2024 Jun 25;10(13):e33196. doi: 10.1016/j.heliyon.2024.e33196 (PMC11263639; doi:10.1016/j.heliyon.2024.e33196)
Supplement: Multimedia component 1 [file mmc1.pptx]

## Slide 1
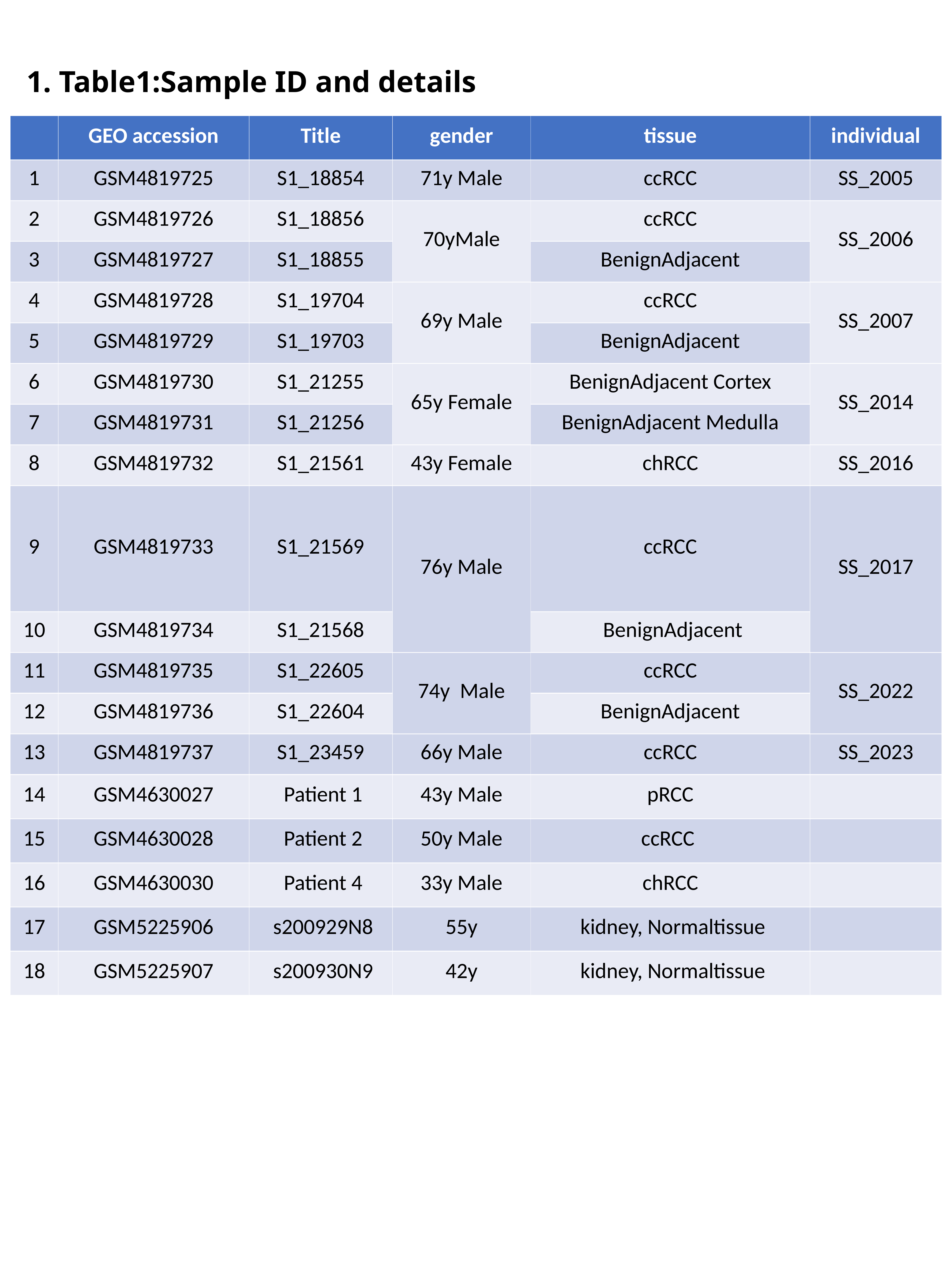

1. Table1:Sample ID and details
| | GEO accession | Title | gender | tissue | individual |
| --- | --- | --- | --- | --- | --- |
| 1 | GSM4819725 | S1\_18854 | 71y Male | ccRCC | SS\_2005 |
| 2 | GSM4819726 | S1\_18856 | 70yMale | ccRCC | SS\_2006 |
| 3 | GSM4819727 | S1\_18855 | | BenignAdjacent | |
| 4 | GSM4819728 | S1\_19704 | 69y Male | ccRCC | SS\_2007 |
| 5 | GSM4819729 | S1\_19703 | | BenignAdjacent | |
| 6 | GSM4819730 | S1\_21255 | 65y Female | BenignAdjacent Cortex | SS\_2014 |
| 7 | GSM4819731 | S1\_21256 | | BenignAdjacent Medulla | |
| 8 | GSM4819732 | S1\_21561 | 43y Female | chRCC | SS\_2016 |
| 9 | GSM4819733 | S1\_21569 | 76y Male | ccRCC | SS\_2017 |
| 10 | GSM4819734 | S1\_21568 | | BenignAdjacent | |
| 11 | GSM4819735 | S1\_22605 | 74y Male | ccRCC | SS\_2022 |
| 12 | GSM4819736 | S1\_22604 | | BenignAdjacent | |
| 13 | GSM4819737 | S1\_23459 | 66y Male | ccRCC | SS\_2023 |
| 14 | GSM4630027 | Patient 1 | 43y Male | pRCC | |
| 15 | GSM4630028 | Patient 2 | 50y Male | ccRCC | |
| 16 | GSM4630030 | Patient 4 | 33y Male | chRCC | |
| 17 | GSM5225906 | s200929N8 | 55y | kidney, Normaltissue | |
| 18 | GSM5225907 | s200930N9 | 42y | kidney, Normaltissue | |

## Slide 2
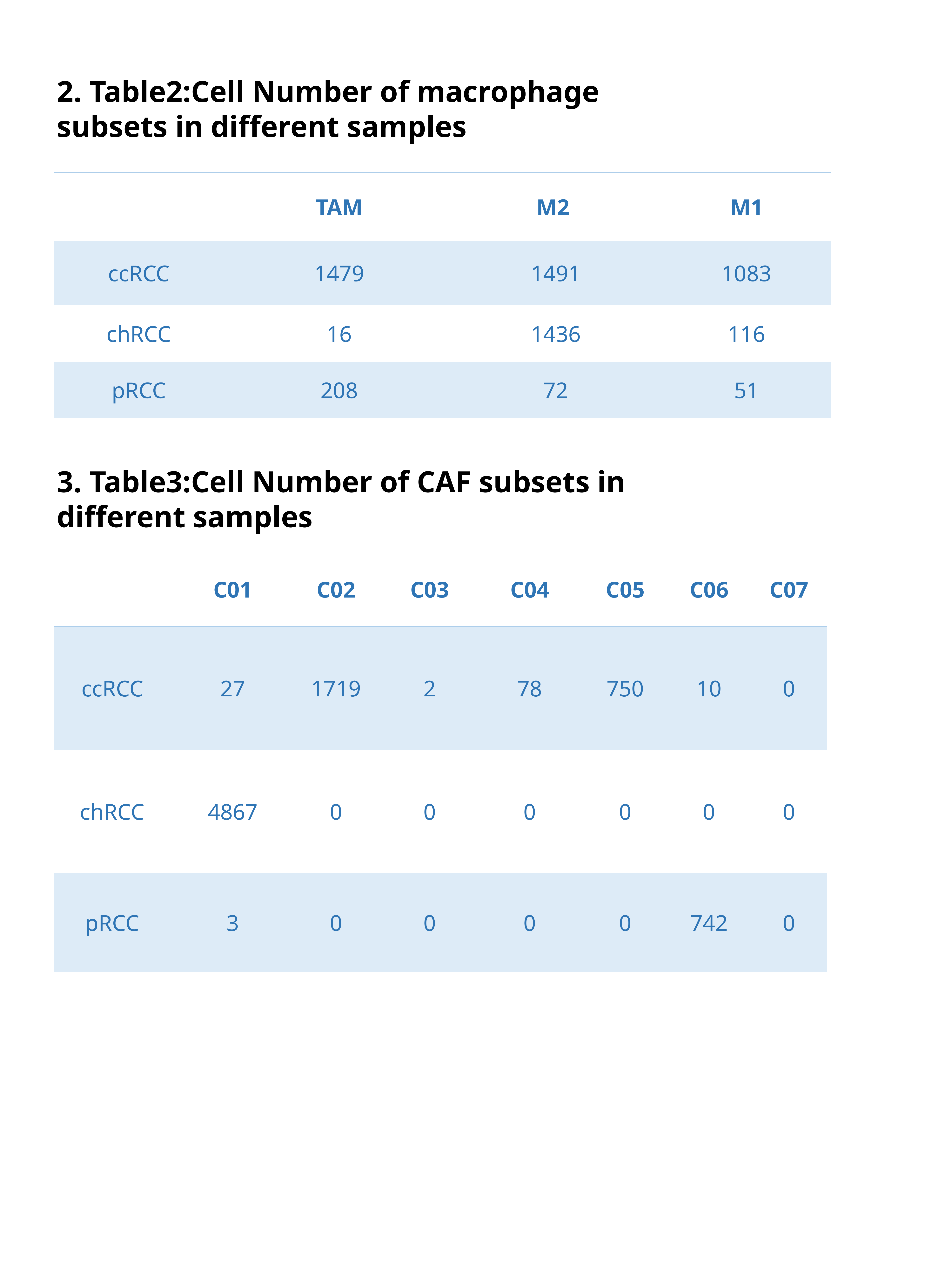

2. Table2:Cell Number of macrophage subsets in different samples
| | TAM | M2 | M1 |
| --- | --- | --- | --- |
| ccRCC | 1479 | 1491 | 1083 |
| chRCC | 16 | 1436 | 116 |
| pRCC | 208 | 72 | 51 |
3. Table3:Cell Number of CAF subsets in different samples
| | C01 | C02 | C03 | C04 | C05 | C06 | C07 |
| --- | --- | --- | --- | --- | --- | --- | --- |
| ccRCC | 27 | 1719 | 2 | 78 | 750 | 10 | 0 |
| chRCC | 4867 | 0 | 0 | 0 | 0 | 0 | 0 |
| pRCC | 3 | 0 | 0 | 0 | 0 | 742 | 0 |

## Slide 3
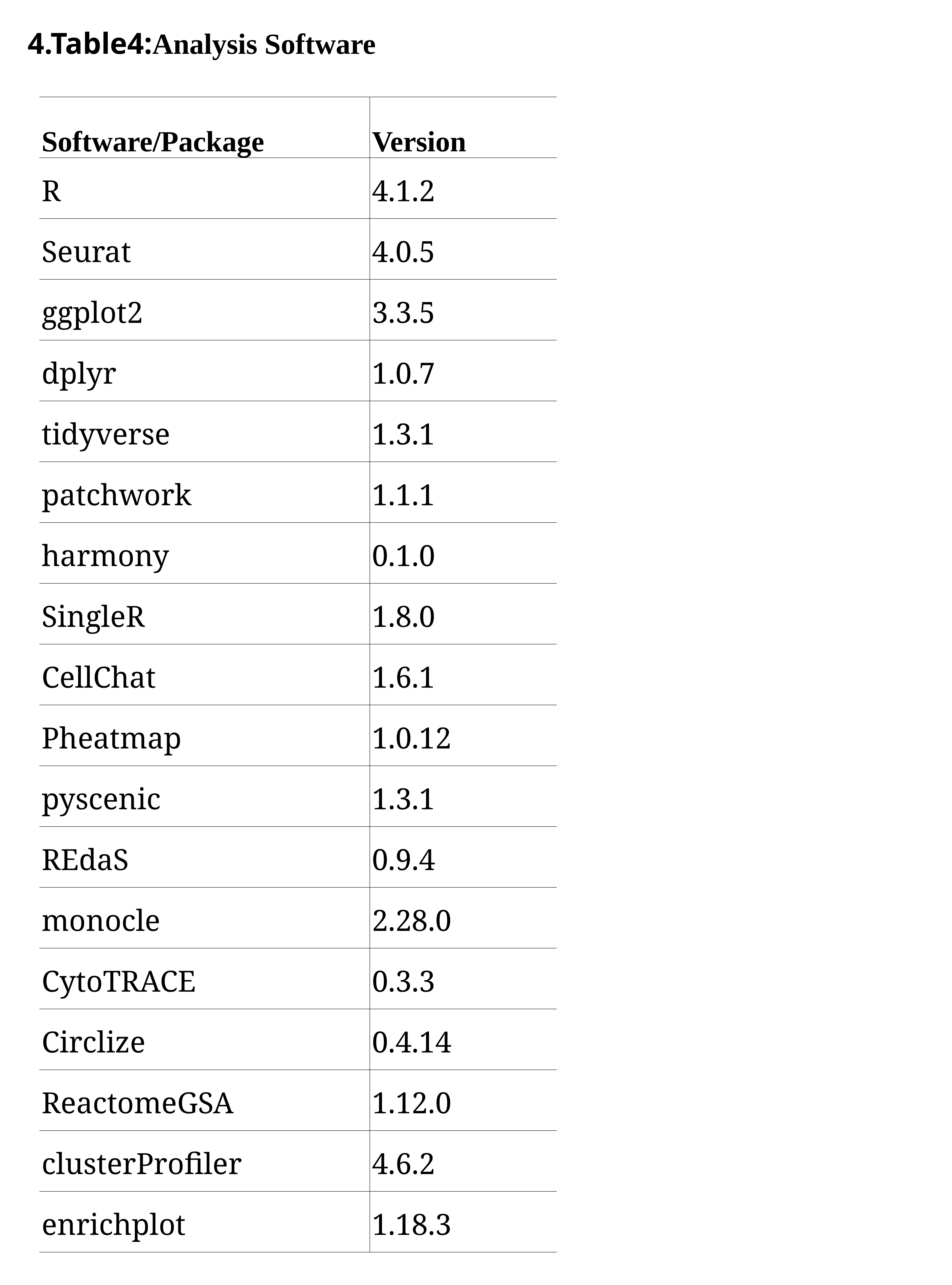

4.Table4:Analysis Software
| Software/Package | Version |
| --- | --- |
| R | 4.1.2 |
| Seurat | 4.0.5 |
| ggplot2 | 3.3.5 |
| dplyr | 1.0.7 |
| tidyverse | 1.3.1 |
| patchwork | 1.1.1 |
| harmony | 0.1.0 |
| SingleR | 1.8.0 |
| CellChat | 1.6.1 |
| Pheatmap | 1.0.12 |
| pyscenic | 1.3.1 |
| REdaS | 0.9.4 |
| monocle | 2.28.0 |
| CytoTRACE | 0.3.3 |
| Circlize | 0.4.14 |
| ReactomeGSA | 1.12.0 |
| clusterProfiler | 4.6.2 |
| enrichplot | 1.18.3 |

## Slide 4
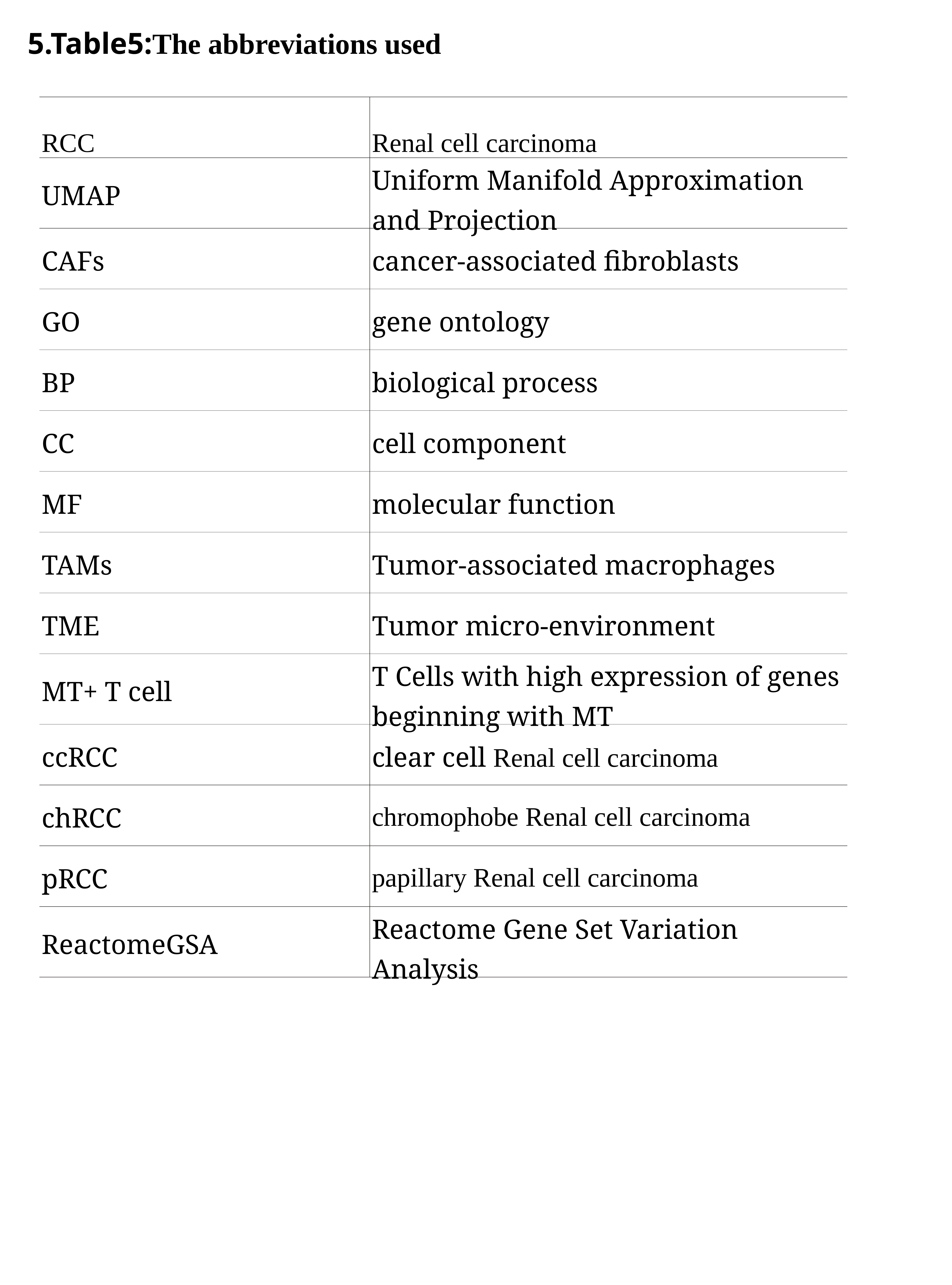

5.Table5:The abbreviations used
| RCC | Renal cell carcinoma |
| --- | --- |
| UMAP | Uniform Manifold Approximation and Projection |
| CAFs | cancer-associated fibroblasts |
| GO | gene ontology |
| BP | biological process |
| CC | cell component |
| MF | molecular function |
| TAMs | Tumor-associated macrophages |
| TME | Tumor micro-environment |
| MT+ T cell | T Cells with high expression of genes beginning with MT |
| ccRCC | clear cell Renal cell carcinoma |
| chRCC | chromophobe Renal cell carcinoma |
| pRCC | papillary Renal cell carcinoma |
| ReactomeGSA | Reactome Gene Set Variation Analysis |

## Slide 5
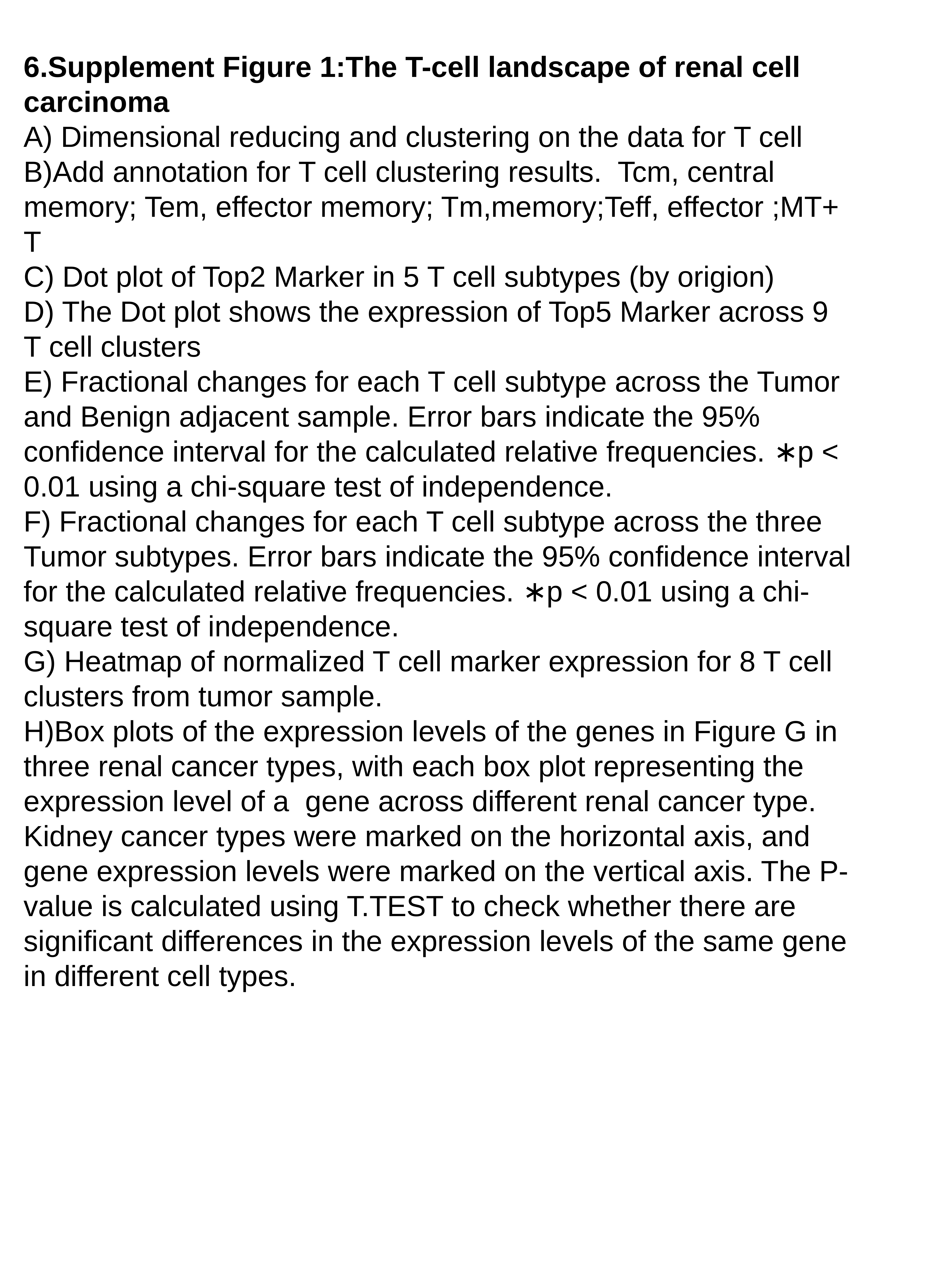

6.Supplement Figure 1:The T-cell landscape of renal cell carcinoma
A) Dimensional reducing and clustering on the data for T cell
B)Add annotation for T cell clustering results. Tcm, central memory; Tem, effector memory; Tm,memory;Teff, effector ;MT+ T
C) Dot plot of Top2 Marker in 5 T cell subtypes (by origion)
D) The Dot plot shows the expression of Top5 Marker across 9 T cell clusters
E) Fractional changes for each T cell subtype across the Tumor and Benign adjacent sample. Error bars indicate the 95% confidence interval for the calculated relative frequencies. ∗p < 0.01 using a chi-square test of independence.
F) Fractional changes for each T cell subtype across the three Tumor subtypes. Error bars indicate the 95% confidence interval for the calculated relative frequencies. ∗p < 0.01 using a chi-square test of independence.
G) Heatmap of normalized T cell marker expression for 8 T cell clusters from tumor sample.
H)Box plots of the expression levels of the genes in Figure G in three renal cancer types, with each box plot representing the expression level of a gene across different renal cancer type. Kidney cancer types were marked on the horizontal axis, and gene expression levels were marked on the vertical axis. The P-value is calculated using T.TEST to check whether there are significant differences in the expression levels of the same gene in different cell types.

## Slide 6
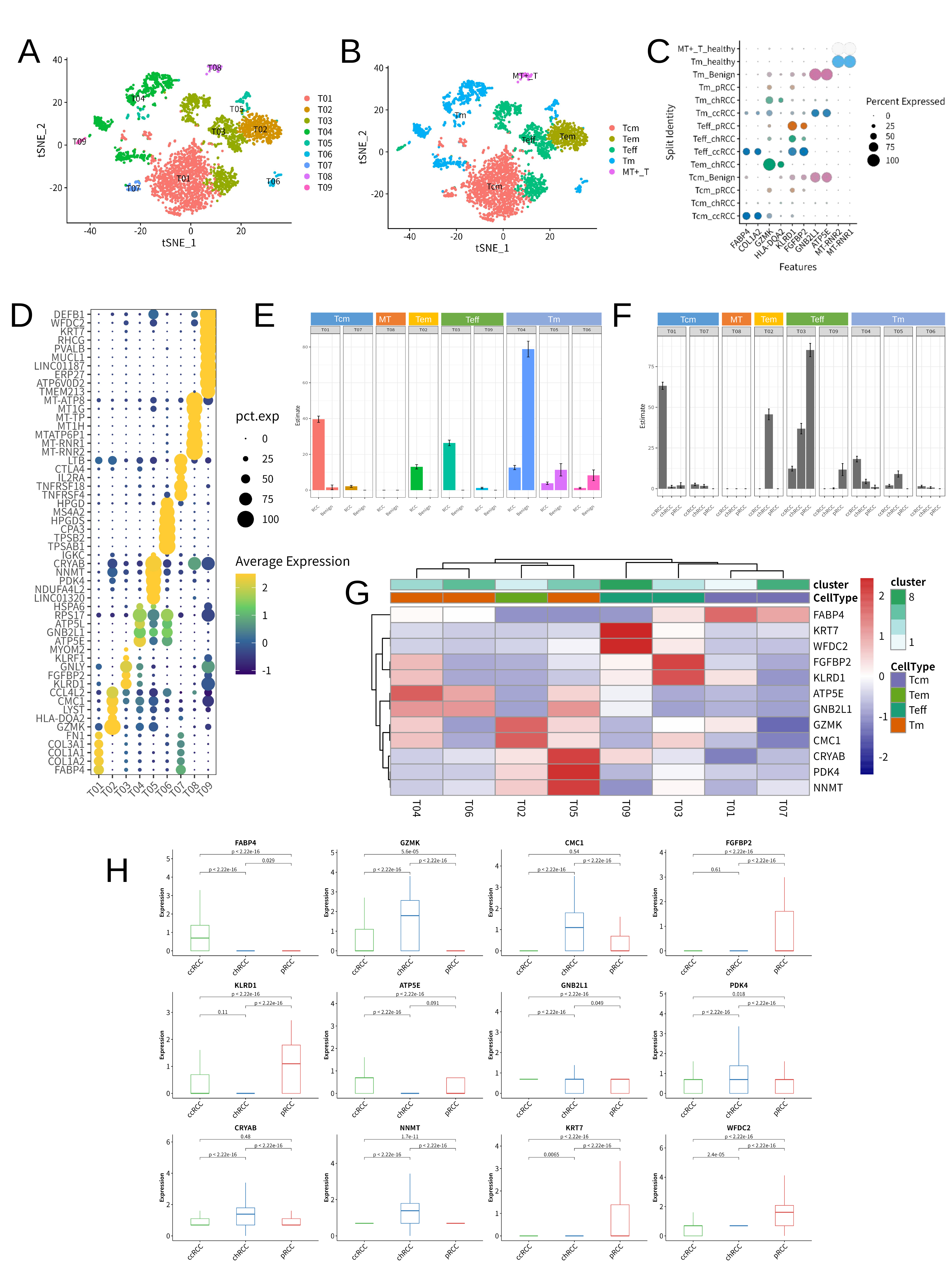

A
B
C
D
E
F
Tcm
MT
Tem
Tm
Teff
Tem
Teff
Tm
Tcm
MT
G
H

## Slide 7
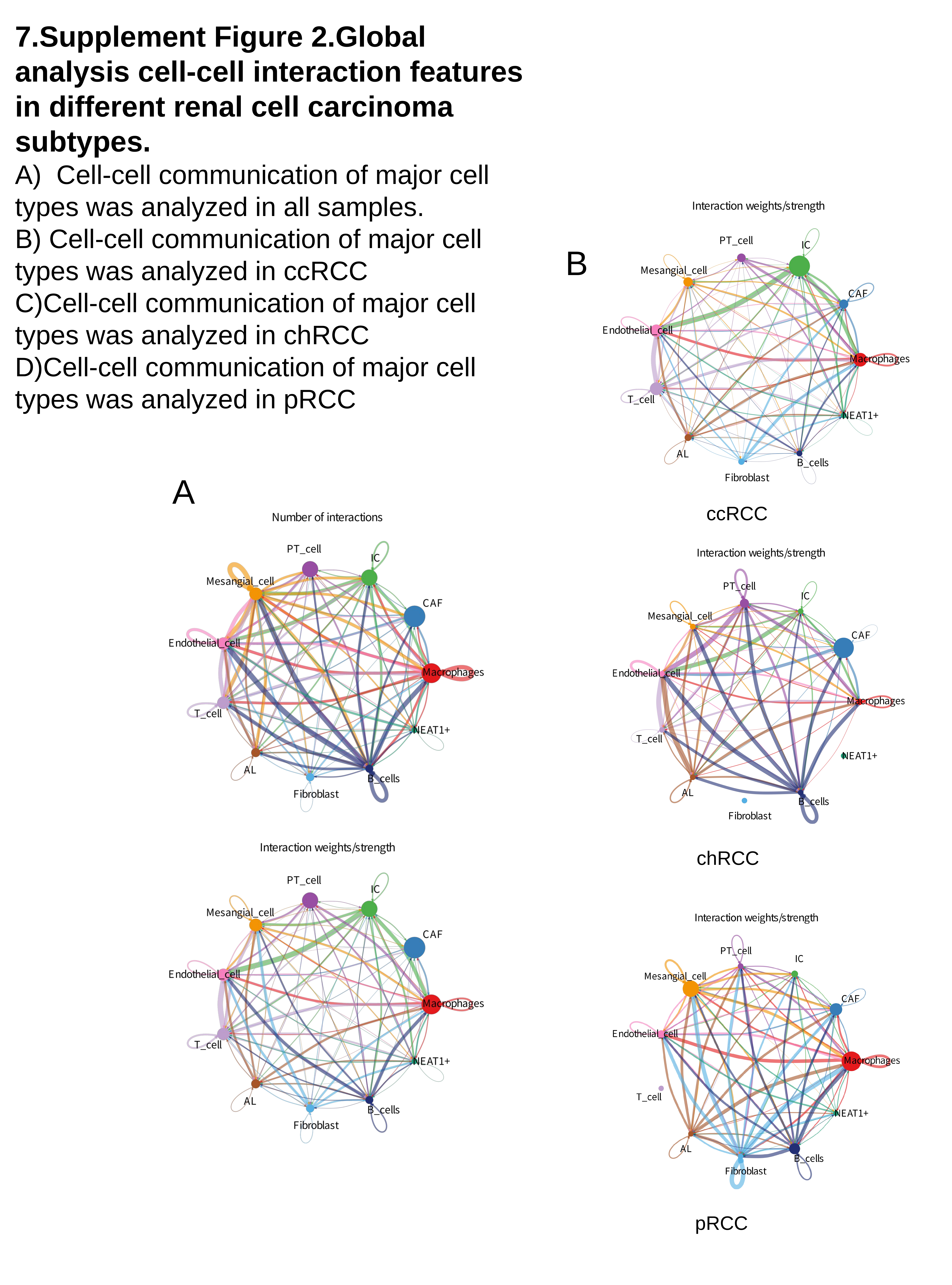

7.Supplement Figure 2.Global analysis cell-cell interaction features in different renal cell carcinoma subtypes.
A) Cell-cell communication of major cell types was analyzed in all samples.
B) Cell-cell communication of major cell types was analyzed in ccRCC
C)Cell-cell communication of major cell types was analyzed in chRCC
D)Cell-cell communication of major cell types was analyzed in pRCC
ccRCC
B
A
chRCC
pRCC
